# Catalytic core function of yeast Pah1 phosphatidate phosphatase reveals structural insight into its membrane localization and activity control

**DOI:** 10.1016/j.jbc.2023.105560

**Published:** 2023-12-12

**Authors:** Gil-Soo Han, Joanna M. Kwiatek, Kam Shan Hu, George M. Carman

**Affiliations:** Department of Food Science and the Rutgers Center for Lipid Research, New Jersey Institute for Food, Nutrition, and Health, Rutgers University, New Brunswick, New Jersey, USA

**Keywords:** lipid, phospholipid, phosphatidate, diacylglycerol, triacylglycerol, phosphadidate phosphatase, Pah1, lipin 1, phosphorylation, dephosphorylation, yeast

## Abstract

The *PAH1*-encoded phosphatidate (PA) phosphatase is a major source of diacylglycerol for the production of the storage lipid triacylglycerol and a key regulator for the *de novo* phospholipid synthesis in *Saccharomyces cerevisiae*. The catalytic function of Pah1 depends on its membrane localization which is mediated through its phosphorylation by multiple protein kinases and dephosphorylation by the Nem1-Spo7 protein phosphatase complex. The full-length Pah1 is composed of a catalytic core (N-LIP and HAD-like domains, amphipathic helix, and the WRDPLVDID domain) and non-catalytic regulatory sequences (intrinsically disordered regions, RP domain, and acidic tail) for phosphorylation and interaction with Nem1-Spo7. How the catalytic core regulates Pah1 localization and cellular function is not clear. In this work, we analyzed a variant of Pah1 (*i.e.*, Pah1-CC (catalytic core)) that is composed only of the catalytic core. Pah1-CC expressed on a low-copy plasmid complemented the *pah1*Δ mutant phenotypes (*e.g.*, nuclear/ER membrane expansion, reduced levels of triacylglycerol, and lipid droplet formation) without requiring Nem1-Spo7. The cellular function of Pah1-CC was supported by its PA phosphatase activity mostly associated with the membrane fraction. Although functional, Pah1-CC was distinct from Pah1 in the protein and enzymological properties, which include overexpression toxicity, association with heat shock proteins, and significant reduction of the *V*_max_ value. These findings on the Pah1 catalytic core enhance the understanding of its structural requirements for membrane localization and activity control.

Phosphatidic acid (PA) phosphatase (PAP), which dephosphorylates PA to diacylglycerol (DAG), plays a major role in the synthesis of the storage lipid triacylglycerol (TAG) by providing its direct precursor (1) (Fig. 1). In *Saccharomyces cerevisiae*, PAP is encoded by four different genes (*i.e.*, *APP1*, *DPP1*, *LPP1*, and *PAH1*) of which *PAH1* is mainly responsible for TAG synthesis (2, 3, 4, 5, 6). The *PAH1*-encoded PAP, which consumes the phospholipid precursor PA, also exerts a negative regulatory control on the *de novo* synthesis of membrane phospholipids (4, 7, 8) (Fig. 1). Accordingly, Pah1 PAP is crucial not only for the production of the storage lipid but also for the regulation of phospholipid synthesis during cell growth. In yeast cells, Pah1 enzyme activity is lower in the exponential phase of growth when the requirement of membrane phospholipids is higher, but increases as cell growth reaches the stationary phase with the accumulation of TAG (5, 9, 10, 11).

**Figure 1:**
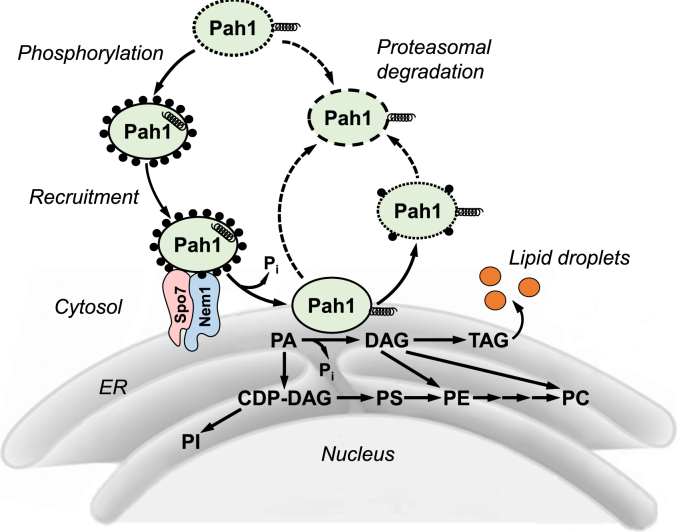
**Model for the regulation of Pah1 PAP and its role in lipid synthesis.** Following its expression, Pah1 is unstable (*dotted line*) and susceptible to proteasomal degradation (indicated by the *dashed line with arrow*). Pah1 is stabilized by its phosphorylation (*black circles*) but is inactive due to its sequestration in the cytosol apart from its substrate PA that resides in the nuclear/ER membrane. The amphipathic helix (*black spiral*) of Pah1 is presumably not exposed in its phosphorylated state. The phosphorylated Pah1 translocates to the nuclear/ER membrane through interaction with the Nem1-Spo7 phosphatase complex, which dephosphorylates Pah1 and presumably exposes its amphipathic helix to permit association with the membrane surface. Dephosphorylated Pah1 catalyzes the dephosphorylation of PA to produce DAG, which is subsequently acylated to form the TAG that is stored in lipid droplets. Unphosphorylated Pah1 or Pah1 phosphorylated by protein kinase C dissociates from the membrane and is subject to proteasomal degradation. Under certain conditions (*e.g.*, choline and/or ethanolamine supplementation), the DAG produced in the reaction may be converted to phosphatidylcholine (*PC*) and/or phosphatidylethanolamine (*PE*) by way of the Kennedy pathway. When Pah1 function is attenuated (*e.g.*, during the exponential phase of growth), the substrate PA is channeled into membrane phospholipids (*e.g.*, phosphatidylserine (*PS*), phosphatidylethanolamine, phosphatidylcholine, and phosphatidylinositol (*PI*)) by way of CDP-DAG. Detailed aspects of this model are reviewed elsewhere (33, 57).

The function of Pah1, which lacks a transmembrane domain, depends on its localization to the membrane where the substrate PA is present. As a peripheral membrane enzyme, Pah1 translocates from the cytosol to the nuclear/ER membrane through its posttranslational modifications, phosphorylation and dephosphorylation (4, 12, 13) (Fig. 1). Pah1 is a highly phosphorylated protein in the cytosol with its phosphorylation catalyzed by multiple protein kinases (14, 15, 16, 17, 18, 19, 20, 21). The phosphorylated form of Pah1 is stable against its proteolysis by the 20S proteasome (22, 23), and dephosphorylated by its protein phosphatase complex Nem1-Spo7 residing in the nuclear/ER membrane (24, 25). Upon its dephosphorylation, Pah1 released from the Nem1-Spo7 complex establishes an interaction with the nuclear/ER membrane and initiates PAP activity (13) (Fig. 1).

Different domains/regions of Pah1 are associated with its catalytic competence and translocation to and interaction with the nuclear/ER membrane. For enzyme function, the HAD-like domain containing the D*X*D*X*(T/V) catalytic motif is predicted to form a catalytic core by interacting with the N-LIP domain (4, 26, 27). The N-terminal amphipathic helix is responsible for interaction with the membrane, facilitating the active site to recognize PA in the membrane (13). The WRDPLVDID domain, which is C-terminal to the HAD-like domain (28), is required for the *in vivo* catalytic function (27, 28). For the nuclear/ER localization of Pah1, the rest of its sequence (*i.e.*, intrinsically disordered regions (IDRs), regulation of phosphorylation (RP) domain, and C-terminal acidic tail) is involved to interact with the organelle-localized Nem1-Spo7 (28, 29, 30). The IDRs contain almost all of the sites of phosphorylation that serve for interaction with the Nem1-Spo7 protein phosphatase. The phosphorylation of many of those sites is regulated by the RP domain (30). In addition to the phosphorylation-mediated interaction of Pah1 with Nem1-Spo7, its acidic tail is required to interact with the protein phosphatase complex (29).

The regulatory sequences of Pah1 for its phosphorylation and interaction with the Nem1-Spo7 complex are crucial for nuclear/ER membrane localization. However, little is known about the effect of the catalytic core of Pah1 on its localization and cellular function. In this work, we constructed and analyzed the variant of Pah1 (*i.e.*, Pah1-CC (catalytic core)) that is composed only of the domains/regions required for *in vivo* catalytic activity. The Pah1 variant, which lacks the sequence for interaction with Nem1-Spo7 and for phosphorylation, was functional and complemented the *pah1*Δ mutant, but showed distinct subcellular localization with altered protein and catalytic properties. These findings advance the mechanistic understanding of Pah1 for its translocation to the nuclear/ER membrane.

## Results

### Pah1-CC is a functional PAP enzyme

**Figure 2:**
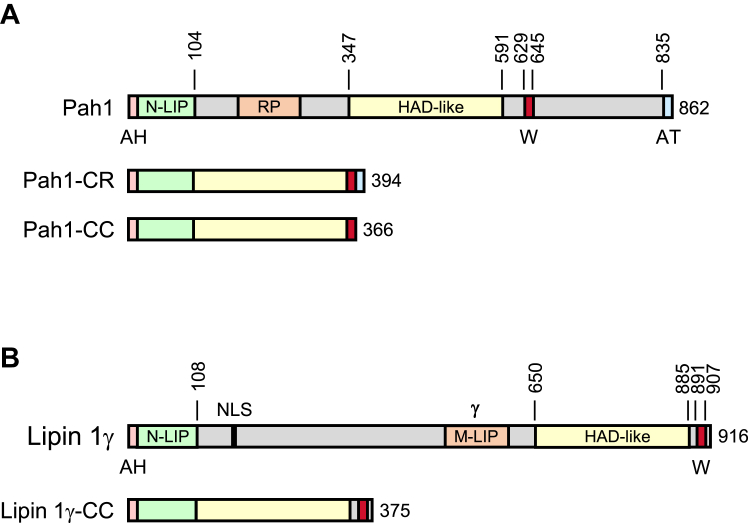
**Schematic diagrams of yeast Pah1 and human lipin 1γ.** The conserved domains and characteristic sequences are shown for yeast Pah1 and its deletion variants (Pah1-CR and Pah1-CC) (*A*), and for human lipin 1γ and its deletion variant lipin 1γ-CC (*B*). The number next to a diagram indicates the total amino acids of the protein, and the number above the diagram indicates the amino acid residues. The *gray-colored regions* represent intrinsically disordered regions. *AH*, amphipathic helix; *AT*, acidic tail; HAD-like, haloacid dehalogenase-like domain; *M-LIP*, middle lipin domain; *N-LIP*, N-terminal lipin domain; *NLS*, nuclear localization signal; *RP*, regulation of phosphorylation domain; *W*, WRDPLVDID domain; γ, isoform-specific sequence.

To gain insight into the molecular architecture of Pah1, we examined the sequence required for *in vivo* function. In a previous study, we showed that Pah1 lacking IDRs, *i.e.*, Pah1-CR (conserved regions) (Fig. 2), is functional as PAP (28). The Pah1 variant is composed of the amphipathic helix, the N-LIP and HAD-like domains, the WRDPLVDID domain containing a conserved tryptophan residue (*i.e.*, Trp-637), and an acidic tail (27, 28). Since Pah1-CR virtually lacks the phosphorylation sites, we asked whether its function requires the C-terminal acidic tail, which is involved in an interaction with the Nem1-Spo7 phosphatase complex for Pah1 dephosphorylation (29). To address this question, we removed the acidic tail sequence from *PAH1-CR* to generate *PAH1-CC* (Fig. 2) and examined the functional roles of the smaller variant. In this work, we included data for the Pah1-CR form for comparison.

**Figure 3:**
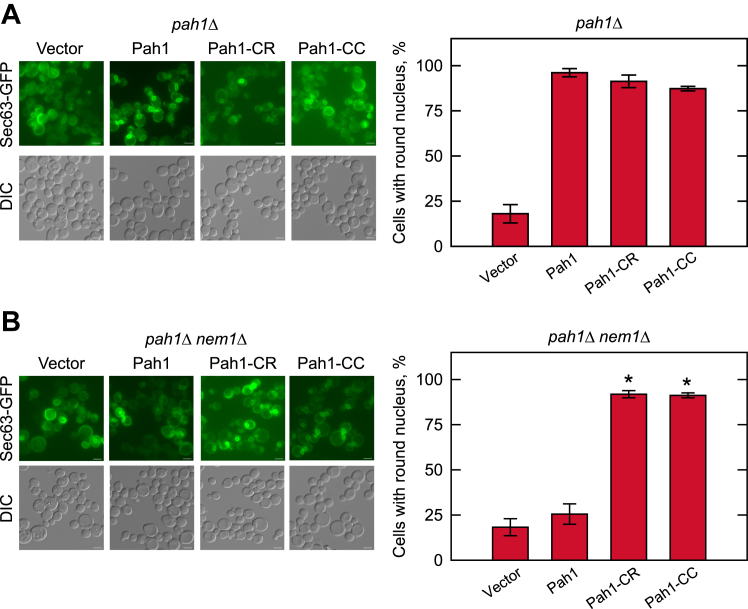
**Nuclear morphology of the *pah1*Δ and *pah1*Δ *nem1*Δ cells expressing Pah1, Pah1-CR, and Pah1-CC.** The *pah1*Δ (SS1026) (*A*) and *pah1*Δ *nem1*Δ (SS1132) (*B*) cells harboring pRS415, pGH315 or its derivative (pGH315-CR or pGH315-CC) along with YCplac33-*SEC63-GFP* were grown at 30 °C to the exponential phase in the SC-Leu-Ura medium. The nuclear/ER reporter Sec63-GFP of the *pah1*Δ and *pah1*Δ *nem1*Δ transformants were visualized by fluorescence microscopy (*left*), and counted for containing a round nucleus (*right*). The scale bar represents 2 μm. The percentage of nuclear morphology was determined from > 3 fields of view (∼300 cells). The data are mean ± SD (*error bars*). *∗p* < 0.05 versus Pah1. *DIC*, differential interference contrast.

The *pah1*Δ mutant, which increases *de novo* phospholipid synthesis due to the lack of PA dephosphorylation, is characterized by nuclear/ER membrane expansion (24). Accordingly, we examined the nuclear morphology of *pah1*Δ cells transformed with *PAH1-CC* and the ER membrane reporter *SEC63-GFP* (Fig. 3*A*). As was expected, fluorescence microscopy showed that the expression of WT Pah1 complemented the phenotype of the *pah1*Δ mutant, resulting in a normal round nuclear morphology. Pah1-CC, as well as Pah1-CR, also corrected the aberrant nuclear morphology (Fig. 3*A*).

**Figure 4:**
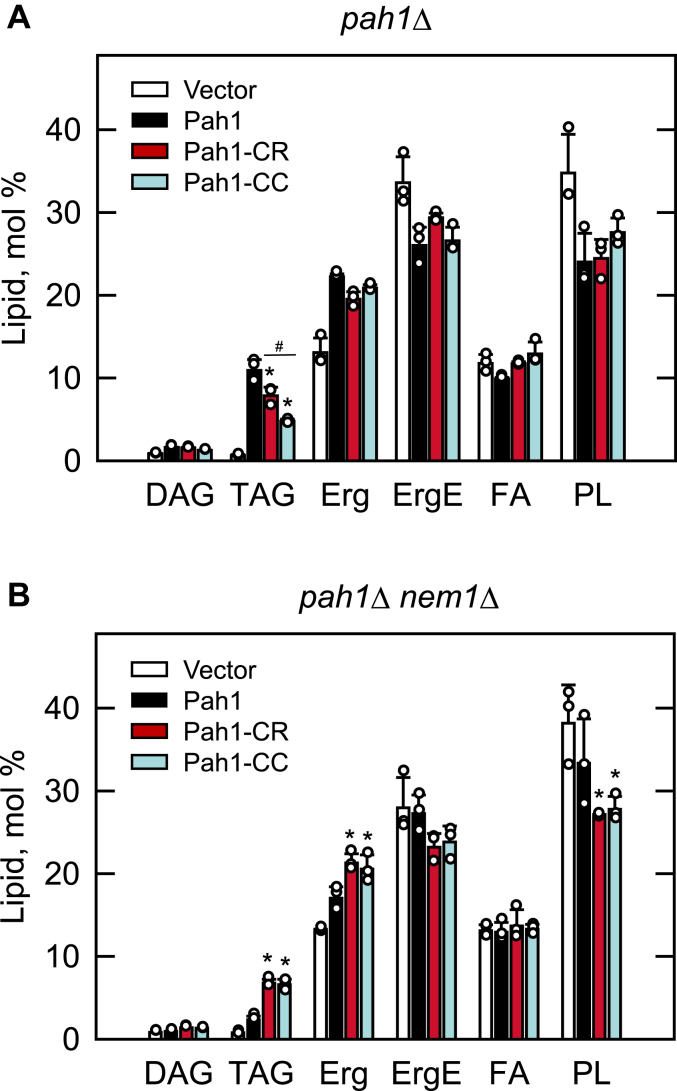
**Lipid composition of the *pah1*Δ and *pah1*Δ *nem1*Δ cells expressing Pah1, Pah1-CR, and Pah1-CC.** The *pah1*Δ (SS1026) (*A*) and *pah1*Δ *nem1*Δ (SS1132) (*B*) cells harboring pRS415, pGH315, or its derivative (pGH315-CR or pGH315-CC) along with YCplac33-*SEC63-GFP* were grown at 30 °C to the stationary phase in the SC-Leu-Ura medium containing [2-^14^C]acetate (1 μCi/ml). Lipids were extracted from the radiolabeled cells and separated by thin-layer chromatography, followed by phosphorimaging and ImageQuant analysis. The data of the *pah1*Δ and *pah1*Δ *nem1*Δ transformants are mean ± SD (*error bars*) from biological triplicates, and the individual data points are shown. *∗p* < 0.05 *versus* Pah1. ^#^*p* < 0.05 of Pah1-CC *versus* Pah1-CR. *Erg*, ergosterol; *ErgE*, ergosterol ester; *FA*, fatty acid; *PL*, phospholipid.

**Figure 5:**
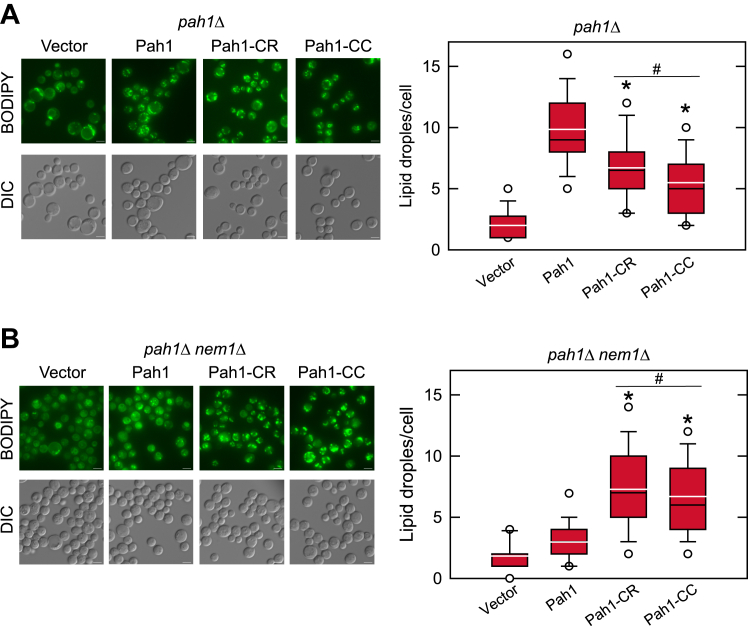
**Lipid droplet formation of the *pah1*Δ and *pah1*Δ *nem1*Δ cells expressing Pah1, Pah1-CR, and Pah1-CC.** The *pah1*Δ (SS1026) (*A*) and *pah1*Δ *nem1*Δ (SS1132) (*B*) cells harboring pRS415, pGH315 or its derivative (pGH315-CR or pGH315-CC) along with YCplac33-*SEC63-GFP* were grown at 30 °C to the stationary phase in the SC-Leu-Ura medium, and incubated with the fluorescent dye BODIPY 493/503. The stained lipid droplets of the *pah1*Δ and *pah1*Δ *nem1*Δ transformants were visualized by fluorescence microscopy (*left*) and quantified (*right*). The scale bar represents 2 μm. The number of lipid droplets per cell was determined from > 3 fields of view (∼300 cells). *Left*, the images shown are representative of multiple fields of view. *Right*, the data are presented by the box plot. The *black* and *white lines* are the median and mean values, respectively, and the *black circles* are the outlier data points of the 5th and 95th percentile. *∗p* < 0.05 versus Pah1. ^#^*p* < 0.05 of Pah1-CC versus Pah1-CR. *DIC*, differential interference contrast.

The defect of the *pah1*Δ mutant in DAG production is reflected by a great reduction in TAG synthesis and lipid droplet formation, as well as an increase in membrane phospholipids (4, 31). To examine whether the expression of Pah1-CC complements these phenotypes, the same yeast strains were grown to the stationary phase in the presence of [2-^14^C]acetate for lipid labeling, and in the absence of the radiolabel, for lipid droplet staining with BODIPY 493/503. The TLC analysis of radiolabeled lipids showed that the -CR and -CC variants of Pah1 restored the TAG level at 72% and 44%, respectively, when compared with the complementary effect of WT Pah1 (Fig. 4*A*). The *pah1*Δ cells expressing the mutant variants of Pah1 also restored WT levels of membrane phospholipids. As described previously (4), the levels of ergosterol and ergosterol ester were lower and higher, respectively, in the *pah1*Δ mutant when compared with the wild-type control. The analysis of stained lipid droplets by fluorescence microscopy showed that like WT Pah1, the -CR and -CC variants of the enzyme complemented, yet to a lesser degree, the defect in lipid droplet formation displayed by *pah1*Δ mutant cells (Fig. 5*A*). The differences in the TAG levels and lipid droplets between the -CR and -CC forms were statistically significant. Taken together, these results indicate that Pah1-CC is sufficient to complement the *pah1*Δ mutant and that the acidic tail is not essential for the function of the deletion variant.

### Pah1-CC function does not require Nem1-Spo7 phosphatase activity

The complementation of the *pah1*Δ mutant by Pah1-CC, which lacks the acidic tail sequence for interaction with Nem1-Spo7, suggests that the deletion variant does not require the protein phosphatase complex for its function on the membrane. To determine this possibility, we examined the function of Pah1-CC expressed in the *pah1*Δ *nem1*Δ mutant (*i.e.*, the *pah1*Δ mutant lacking the Nem1-Spo7 complex (12)), with respect to nuclear morphology (Fig. 3*B*), TAG levels (Fig. 4*B*), and lipid droplet formation (Fig. 5*B*). In the absence of the protein phosphatase complex, the expression of the WT Pah1 did not rescue the *pah1*Δ phenotypes of the abnormal nuclear/ER morphology, reduced and elevated levels of TAG and phospholipids, respectively, and the reduced lipid droplets, confirming the importance of the Nem1-Spo7 complex in the regulation of Pah1 function (14, 24, 25). However, the Pah1-CC form of the enzyme complemented the *pah1*Δ mutant phenotypes (Figs. 3*B*, 4*B*, and 5*B*). Additionally, the -CR variant complemented these phenotypes. Thus, Pah1-CR and Pah1-CC, which possesses and lacks the acidic tail, respectively, do not require the Nem1-Spo7 phosphatase activity for their function.

### PAP activity of Pah1-CC is mainly associated with the membrane

**Figure 6:**
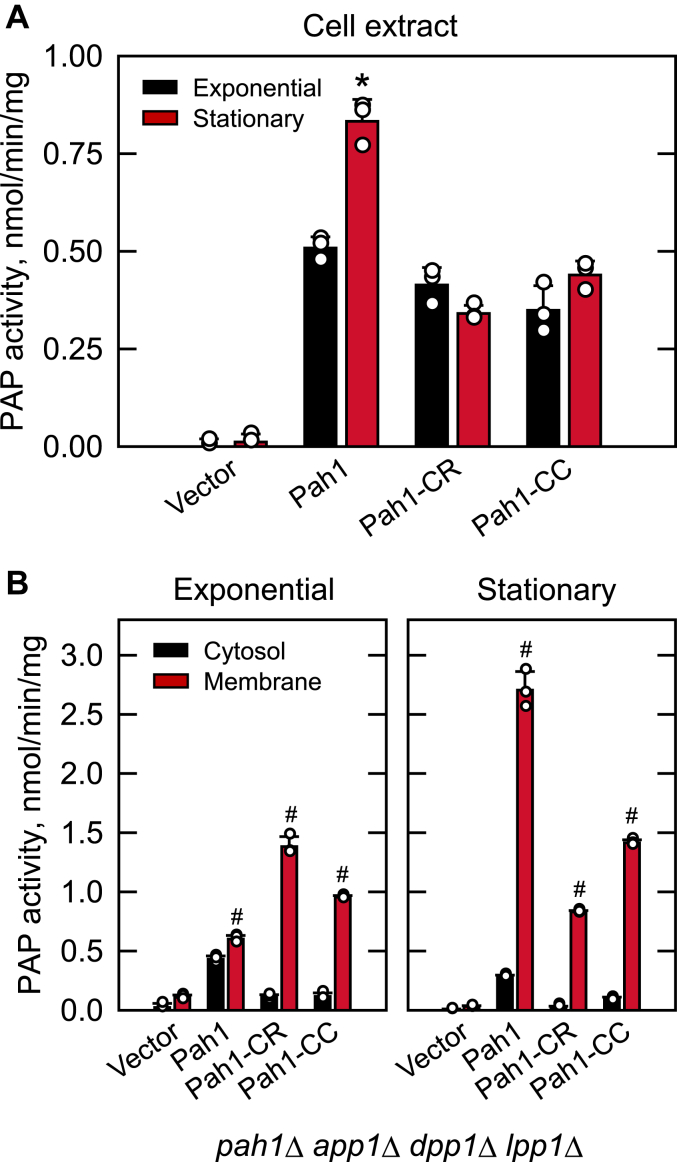
**PAP activity of the *pah1*Δ and *pah1*Δ *nem1*Δ cells expressing Pah1, Pah1-CR, and Pah1-CC.** The *pah1*Δ *app1*Δ *dpp1*Δ *lpp1*Δ (GHY66) cells were transformed with pRS415, pGH315 or its derivative (pGH315-CR or pGH315-CC). The yeast transformants were grown at 30 °C in SC-Leu medium to the exponential and stationary phases, followed by the preparation of cell extracts and the subcellular fractionation into cytosol and total membranes. The PAP activity of the cell extracts (*A*) and subcellular fractions (*B*) was measured with [^32^P]PA. The data are mean ± SD (*error bars*) from triplicate determinations. *∗p* < 0.05 *versus* exponential phase, ^*#*^*p* < 0.05 *versus* cytosol.

We examined the PAP activity from cells expressing the -CR and -CC variant forms of Pah1. To eliminate interference from the PAP activities encoded by the *APP1* (5), *DPP1* (2), and *LPP1* (3) genes, the WT and variant forms of Pah1 were expressed in the *pah1*Δ *app1*Δ *dpp1*Δ *lpp1*Δ quadruple mutant (5). The cells were grown in a synthetic selection medium to the exponential and stationary phases, and cell extracts were prepared to measure the enzyme activity (Fig. 6*A*). As described previously (5), the WT Pah1 PAP activity in the stationary phase of growth was 1.7-fold greater when compared with the PAP activity in the exponential phase. Pah1-CR and -CC exhibited 20 to 30% lower PAP activity in the exponential phase when compared with the same growth phase of WT Pah1. Moreover, unlike the PAP activity of the WT, which was increased in the stationary phase, the deletion variant activities showed little change from the exponential to the stationary phase of growth. The PAP activity from the stationary phase cells expressing the Pah1-CR and -CC variants was 1.9- to 2.4-fold lower than the activity from cells expressing the WT enzyme at the same growth phase.

Considering that Pah1 function requires its membrane localization, we measured the PAP activity from the cytosolic and membrane fractions of the cell (Fig. 6*B*). In the exponential phase, the PAP activity of WT Pah1 was found in the cytosolic and membrane fractions at similar levels. In contrast, the Pah1-CR and -CC PAP activities were mostly associated with the membrane fraction; the membrane-associated activities of the Pah1 variants were eight- to 13-fold greater when compared with their cytosol-associated activities. Moreover, in the exponential phase, the membrane-associated PAP activities of the variants were 1.7- to 2.3-fold greater than the membrane-associated WT enzyme.

In the stationary phase, the membrane-associated PAP activity of the WT enzyme was highly increased with the reduction of the cytosol-associated activity; the WT membrane-associated PAP activity was 9.6-fold higher when compared with the cytosol-associated WT activity. For the Pah1-CR and -CC variants, the PAP activities in the stationary phase were mainly associated with the membrane fraction; the membrane-associated activities were 15- to 25-fold greater when compared with the cytosol-associated activities. Yet, in the stationary phase, the membrane-associated PAP activities of the Pah1-CR and -CC variants were 1.9- to 3.3-fold lower when compared with the membrane-associated WT enzyme.

### Overexpression of Pah1-CC is lethal

**Figure 7:**
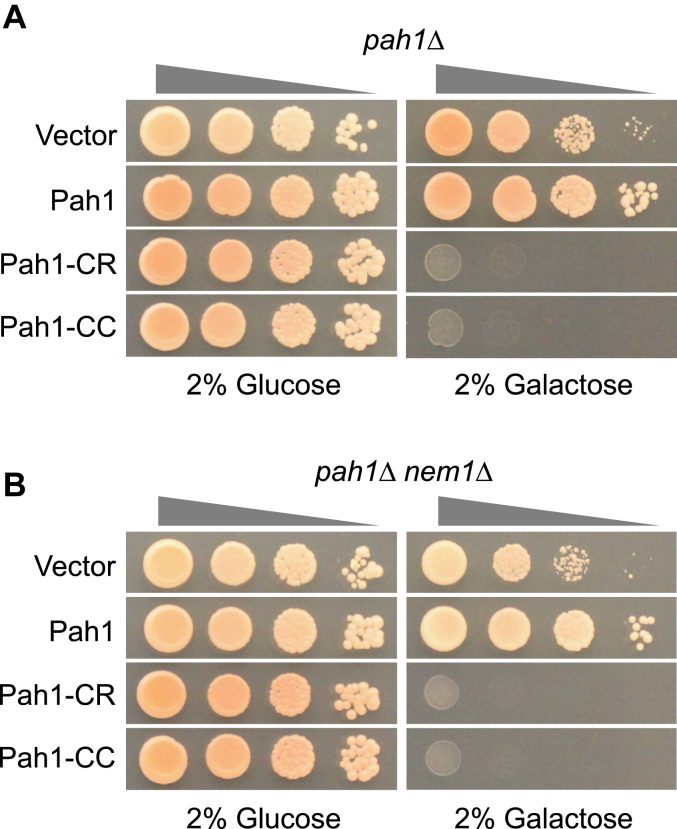
**Overexpression of Pah1-CC causes lethality.** The *pah1*Δ (SS1026) (*A*) and *pah1*Δ *nem1*Δ (SS1132) (*B*) cells were transformed with pYES2, pGH452 or its derivative (pGH473 or pGH465). The yeast transformants were grown overnight to saturation in SC-Ura medium containing glucose as a carbon source. The transformant cultures were washed with water and adjusted to A_600_ = 0.67, followed by 10-fold serial dilution. The diluted cultures (5 μl) were spotted onto SC-Ura medium containing 2% glucose or 2% galactose. The cell growth was scored after incubation for 3 days at 30 °C.

Excess PAP activity would deplete PA required for *de novo* phospholipid synthesis, but accumulate DAG that has an adverse effect on cell growth (32). Accordingly, we examined whether the overexpression of Pah1-CC has any effect on cell growth. For this purpose, we constructed *PAH1-CC* on a multi-copy plasmid under the control of the *GAL1* promoter. The *pah1*Δ mutant transformed with the *PAH1-CC* plasmid was maintained in a synthetic selection medium with glucose as a carbon source and then grown on the galactose-containing medium for the gene overexpression (Fig. 7). Compared with the overexpression of WT Pah1, which had little effect on cell growth, the overexpression of Pah1-CR and -CC, which bypassed the Nem1-Spo7 phosphatase requirement for Pah1 function (Figure 3, Figure 4, Figure 5), had a strong effect in abolishing cell growth upon the galactose induction of the genes. This result indicated that the overexpression of Pah1-CC is detrimental to cell growth.

### Purification, PAP enzyme kinetics, and phosphorylation of Pah1-CC

**Figure 8:**
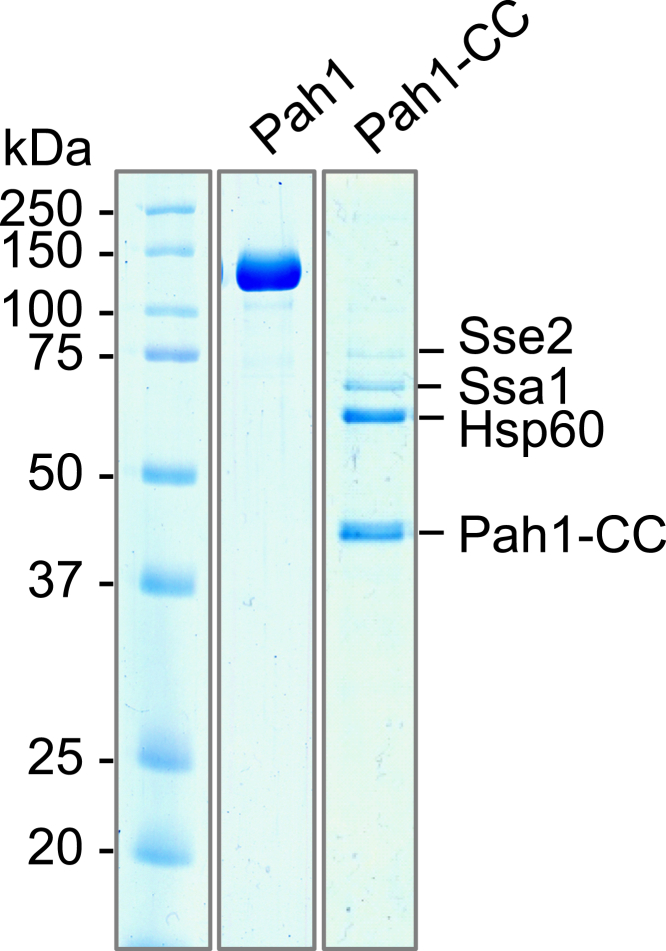
**Purification ofPah1 and Pah1-CC from yeast.** The *pah1*Δ *nem1*Δ (SS1132) cells were transformed with pGH452 or pGH465. The yeast transformants were grown and induced 14 h for the overexpression of Pah1-PtA and Pah1-CC-PtA. Following the induction, the enzymes were affinity-purified and removed from the protein A tag as described in “Experimental procedures.” Purified Pah1 and Pah1-CC were resolved by SDS-PAGE (10% polyacrylamide gel) and stained with Coomassie Blue. The proteins co-purified with Pah1-CC and identified by LC-MS/MS are indicated.

To gain further insight into the Pah1-CC variant of Pah1, the protein was expressed in the *pah1*Δ *nem1*Δ mutant (to facilitate hyperphosphorylation (14)) as a fusion protein with protein A followed by its purification by IgG-Sepharose affinity chromatography, removal of the protein A tag, and anion exchange chromatography as described previously for WT Pah1 (27). An SDS-polyacrylamide gel of the purified WT and mutant proteins is shown in Figure 8. While this purification scheme results in a near homogeneous preparation of WT Pah1, the preparation of the Pah1-CC contained three major contaminants. Liquid chromatography/tandem mass spectrometry (LC-MS/MS) analysis from trypsin digestion of the contaminating proteins revealed their identity to be heat shock proteins Sse2, Ssa1, and Hsp60 (Fig. 8, Table S1).

**Figure 9:**
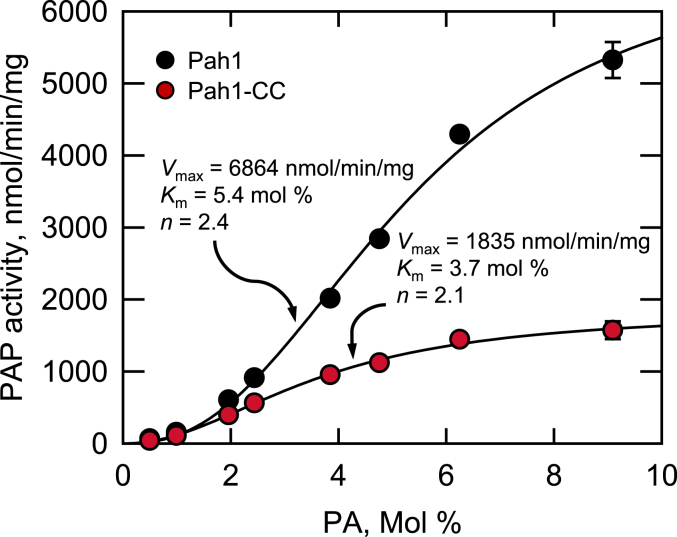
**PAP activity of purified Pah1 and Pah1-CC.** Purified Pah1 and Pah1-CC were measured for PAP activity with 0.2 mM [^32^P]PA by varying its surface concentration with Triton X-100. The data are mean ± SD (*error bars*) from triplicate determinations. *V*_max_, *K*_m_, and Hill number (*n*) values were determined with the Enzyme Kinetics module of SigmaPlot software.

The PAP activity of the purified Pah1 and Pah1-CC enzymes was measured with respect to the surface concentration of PA within Triton X-100/PA-mixed micelles (4). Both forms of the enzyme followed positive cooperative kinetics with respect to the PA surface concentration (Fig. 9). The *V*_max_ for the Pah1-CC enzyme (1835 nmol/min/mg) was 3.7-fold lower than that for the WT enzyme (6864 nmol/min/mg), whereas the *K*_m_ and Hill numbers for both forms of the enzyme were not majorly different.

Pah1 is phosphorylated on multiple sites that are primarily located within the IDRs of the protein (33). Yet, some phosphorylation sites are contained within the N-LIP and HAD-like domains (33). We questioned whether Pah1-CC was subject to endogenous phosphorylation. To permit maximum phosphorylation *in vivo*, Pah1-CC was expressed and purified from the cells lacking the Nem1-Spo7 protein phosphatase (*i.e.*, *pah1*Δ *nem1*Δ mutant) (12, 14). The purified Pah1-CC was subjected to phosphorylation site analysis by LC-MS/MS (Table S1). This analysis revealed that Pah1-CC was phosphorylated on Ser-511, a casein kinase I phosphorylation site contained within the HAD-like domain of the protein.

### Human lipin 1γ-CC complements the temperature-sensitive phenotype of the pah1Δ nem1Δ mutant

**Figure 10:**
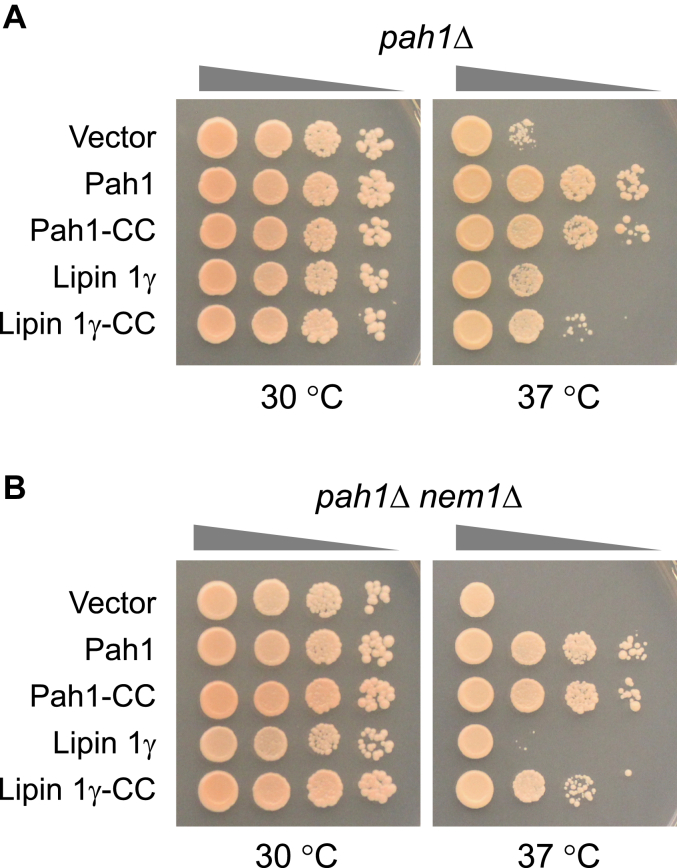
**Lipin 1γ-CC complements the temperature sensitivity of the *pah1*Δ mutant independently of Nem1-Spo7.** The *pah1*Δ (SS1026) (*A*) and *pah1*Δ *nem1*Δ (SS1132) (*B*) cells were transformed with pRS415, pGH315, pGH315-CC, pGH319-ΔHA, or pGH319-CC-ΔHA. The yeast transformants were grown overnight to saturation in SC-Leu medium, and cell density of the cultures was adjusted to A_600_ = 0.67, followed by 10-fold serial dilution. The diluted cultures (5 μl) were placed onto SC-Leu medium and incubated at 30 °C and 37 °C. The temperature sensitivity of cell growth was scored after incubation for 3 days.

Lipin 1, a mammalian ortholog of Pah1 (34) with PAP activity (35, 36), is also comprised of an amphipathic helix, the N-LIP and HAD-like catalytic domains, and a sequence containing the conserved tryptophan (Trp-873) residue (Fig. 2*B*). Like Pah1, lipin 1 is phosphorylated on multiple residues to control its subcellular location (7, 37, 38, 39, 40) and dephosphorylated by an analogous Nem1-Spo7 complex known as CTDNEP1-NEP1R1 (41). Unlike Pah1, lipin 1 has one long IDR sequence between the conserved N-LIP and HAD-like domains for which nearly all phosphorylation sites reside (7, 42) and lacks the acidic tail (Fig. 2*B*). To determine whether human lipin 1γ (one of three splice variants of lipin 1 (36)) lacking the IDR (*i.e.*, lipin 1γ*-*CC) is functional independently of the phosphorylation-mediated regulation, we analyzed the deletion variant expressed under the control of the *PAH1* promoter. Using the temperature sensitivity phenotype of the *pah1*Δ mutant, a striking phenotype that is used to score Pah1 function (4, 24), we examined the effect of lipin 1γ-CC expression in the *pah1*Δ and *pah1*Δ *nem1*Δ mutants (Fig. 10). The *pah1*Δ mutant expressing lipin 1γ showed a poor growth at 37 °C whereas the mutant expressing lipin 1γ-CC showed a little better growth. However, the complementation effect of lipin 1γ-CC was weaker when compared with that of Pah1 or Pah1-CC. In the *pah1*Δ *nem1*Δ mutant, which lacks the Nem1-Spo7 complex (12), we observed that wild-type Pah1 complemented the temperature sensitivity imparted by the *nem1*Δ mutation (12). That Pah1 function was attenuated, but not totally lost with respect to nuclear membrane morphology (Fig. 3*B*), TAG synthesis (Fig. 4*B*), and lipid droplet formation (Fig. 5*B*) indicated that some threshold of Pah1 function is sufficient to permit growth at the elevated temperature in the *nem1*Δ mutant background. The full-length lipin 1γ was non-functional in the *pah1*Δ *nem1*Δ mutant whereas lipin 1γ-CC showed a complementation effect similar to that shown in the *pah1*Δ mutant. These results suggest that lipin 1γ-CC is also functional without the localization control through its phosphorylation and dephosphorylation.

## Discussion

In this work, we showed that Pah1-CC, a variant of Pah1 containing the sequences only for the catalytic core (*i.e.*, amphipathic helix and the N-LIP, HAD-like, and WRDPLVDID domains), complemented the *pah1*Δ mutant for TAG synthesis, lipid droplet formation, nuclear membrane morphology, and temperature sensitivity. Its localization, protein, and enzymological properties were distinct from those of the full-length Pah1. The Pah1-CC characteristics provide a mechanistic insight into the role of the non-catalytic sequences (*e.g.*, IDR, RP domain, and acidic tail) for the nuclear/ER membrane localization of the catalytic core.

The Nem1-Spo7 complex-independent function of Pah1-CC, which complements the *pah1*Δ mutant, indicates that the catalytic core of Pah1 is sufficient for PAP activity on the nuclear/ER membrane. In the subcellular fractionation of cell extracts, the PAP activity of Pah1-CC was mostly associated with the membrane fraction. These results demonstrate that Pah1-CC readily associates with cellular membranes, indicating that the amphipathic helix required for membrane interaction is exposed in the Pah1-CC variant. The nuclear/ER membrane translocation of Pah1 through its phosphorylation and dephosphorylation suggests that the amphipathic helix is not exposed in the phosphorylated state of the enzyme, but exposed by Pah1 dephosphorylation (13). Since the IDR sequence of Pah1 contains almost all of the phosphorylation sites, its phosphorylated residues are presumably responsible for the inhibitory effect on the amphipathic helix. Most probably, the negatively charged phosphates from the phosphorylation would interact with the positively charged lysine or arginine residue of the amphipathic helix (29), and thereby prevent its exposure to membrane interaction. The activation of the amphipathic helix through the dephosphorylation of the IDR sequence has been implicated by the phosphorylation-deficient variants of Pah1 (*e.g.*, Pah1-7A and Pah1-ΔRP) that do not require the Nem1-Spo7 activity to complement the *pah1*Δ mutant (13, 14, 30). Moreover, the AlphaFold-predicted structure of Pah1, which is an unphosphorylated form, shows that the amphipathic helix is exposed outward at the N-terminal end of the N-LIP domain (27, 43, 44). Thus, the amphipathic helix of Pah1-CC, which lacks the IDR phosphorylation sites, would always be in an exposed state for interaction with the membrane.

Based on available information, we postulate the following events for the phosphorylation/dephosphorylation-mediated regulation of Pah1 localization. The phosphorylation-mediated masking of the amphipathic helix is required for Pah1, which lacks a membrane targeting sequence, to associate specifically with the nuclear/ER membrane (13). Phosphorylated Pah1 in the cytosol cannot associate with the membrane due to the masking of the amphipathic helix, but it interacts with the Nem1-Spo7 phosphatase complex through its C-terminal acidic tail and phosphorylated residues (29). The acidic tail, which is rich in negatively charged amino acids, interacts with Nem1-Spo7 (29), possibly through ionic interaction with the positively charged amino acids of the protein phosphatase complex. The phosphorylated residues of Pah1 are recognized by the Nem1-Spo7 complex as the substrates of the protein phosphatase (14, 24, 25, 45). Through the protein-protein interaction, phosphorylated Pah1 in the cytosol would localize to the surface of the nuclear/ER membrane where the Nem1-Spo7 complex resides. Accordingly, its subsequent dephosphorylation by the Nem1-Spo7 complex should result in the exposure of the amphipathic helix. Dephosphorylated Pah1, which is released from the protein phosphatase complex, may associate with the nuclear/ER membrane. The control of the amphipathic helix coupled with the protein-protein interaction ensures that Pah1 translocates from the cytosol specifically to the nuclear/ER membrane (13, 14, 45) (Fig. 1).

Pah1-CC, which is deficient in IDR sequences, is shown to be phosphorylated on Ser-511, a residue located in the HAD-like domain (33). The serine residue is one of eight serine residues phosphorylated by casein kinase I (20). The significance of Pah1-CC phosphorylation on this serine residue is not yet clear and needs an additional study. None of the additional phosphorylation sites that are contained within the N-LIP and HAD-like domains of full-length Pah1 (33) were identified in Pah1-CC. This might be attributed to the hierarchical nature of the phosphorylations in Pah1 (33) and almost all of the phosphorylations are missing because of the IDR deletions in this Pah1 variant.

The yield of Pah1-CC purified from yeast was much lower than that of Pah1, and the purified protein was associated with heat shock proteins Sse2, Ssa1, and Hsp60. In addition, the catalytic core Pah1 composed only of N-LIP and HAD-like domains is poorly soluble and aggregates to form an inclusion body when expressed in *Escherichia coli* (46). These findings suggest that the Pah1-CC, which lacks the non-catalytic sequence, has low solubility and tends to aggregate. The low solubility and aggregation tendency of Pah1-CC could be a reason for its low catalytic activity measured in the PAP assay. The enzyme activity of Pah1 is generally higher in the unphosphorylated state than in the phosphorylated state (14, 15, 16, 18, 19, 21). Accordingly, Pah1-CC, which virtually lacks all phosphorylation sites, was expected to show a higher PAP activity. However, its *V*_max_ was much lower than that of phosphorylated Pah1, but without a significant difference in the *K*_m_ value. A possible reason for the low catalytic activity of Pah1-CC is related to the actual amount of the enzyme available for catalysis. The self-aggregation of Pah1-CC or its association with other proteins reduces the level of the catalytically competent molecules resulting in a decrease in *V*_max_ without affecting *K*_m_. The low solubility of Pah1-CC is also estimated from its amino acid sequence by solubility prediction calculations (47). In this analysis, Pah1-CC is less soluble due to the abundance of hydrophobic amino acids whereas the IDRs and acidic tail of the non-catalytic sequence are highly soluble. The catalytic core of Pah1 that functions in the hydrophobic environment should be stable in the cytosol until it associates with the membrane. The intrinsically disordered, highly charged proteins fused to poorly soluble proteins serve as solubility enhancers by creating a large favorable surface area for water interactions as well as large excluded volumes around the partner proteins (48). Thus, the IDR sequences of Pah1, which are highly soluble, would enhance the solubility of its catalytic core, preventing protein aggregation in the cytosol.

The overexpression of Pah1-CC (or Pah1-CR) caused cell lethality. A similar lethal effect has been shown by the overexpression of the phosphorylation-deficient Pah1 (*e.g.*, Pah1-7A) (14, 15). These findings indicate that the excess of PAP activity that is not controlled by the Nem1-Spo7 complex is detrimental to cell growth and viability. Too high PAP activity would deplete PA that is required for the *de novo* synthesis of membrane phospholipids and thus for cell proliferation (14, 15). In addition, the accumulation of the product DAG would affect cell viability (32) presumably by disrupting the lamellar phase of cellular membranes (49) and endomembrane homeostasis (50). The Nem1-Spo7-dependent membrane association of Pah1 limits its amount on the nuclear/ER membrane by the protein phosphatase activity, which has the effect of preventing the detrimental accumulation of PAP activity on the membrane.

The discovery that the *S. cerevisiae PAH1* is the gene encoding the Mg^2+^-dependent PAP that controls the bifurcation of PA for the synthesis of TAG or membrane phospholipids led to the revelation that human lipin 1 is a PAP enzyme (4). The conservation of the catalytic cores of yeast Pah1 and human lipin 1 provides the impetus that yeast PAP is a good model for studying the enzyme in higher eukaryotes. This may be true when it comes to the mode of action and kinetics of activity, but some aspects of these enzymes differ. On one hand, the acidic tail that is required for Pah1 interaction with the Nem1-Spo7 complex (29) and the RP domain located between the N-LIP and HAD-like domains that control the phosphorylation of Pah1 (30) are not conserved in mammalian lipins. On the other hand, the M-LIP domain found within the large IDR of lipin 1 that is important for its dimerization and membrane association (51) is not found in Pah1. Differences between the yeast and human PAP enzymes are reflected in the observation that full-length human lipin 1 does not fully complement the temperature sensitivity imparted by the *S. cerevisiae pah1*Δ mutation (52). As in yeast, the localization of lipin 1 in mammalian cells is controlled by its phosphorylation by multiple protein kinases within its IDR (7, 37, 38, 39, 40, 41) and its dephosphorylation by the CTDNEP1-NEP1R1 protein phosphatase complex (41). However, the phosphorylation sites in lipin 1 differ from those in Pah1 (7, 42) and their phosphorylation in yeast cells may not be recognized by the Nem1-Spo7 complex. Moreover, the lack of the acidic tail compromises lipin 1 for its interaction with the Nem1-Spo7 complex at the nuclear/ER membrane. Thus, the components/attributes necessary for lipin 1 to be functional in yeast are absent. However, the lipin 1γ-CC, which lacks the IDR with its phosphorylation sites, could bypass the Nem1-Spo7 complex requirement for membrane interaction and permit complementation of the temperature sensitivity caused by the *pah1*Δ mutation. The lipin 1γ-CC construct should prove useful for mechanistic aspects of the PAP enzyme in mammalian systems.

Overall, the work presented here advances our understating of the structural constituents of Pah1 (*i.e.*, catalytic core and non-catalytic regulatory sequences) and the enzyme posttranslational modification for its translocation from the cytosol to the nuclear/ER membrane for catalytic function.

## Experimental procedures

### Reagents

All chemicals were reagent grade. Culture medium components were from BD Difco. DNA purification kits were from Qiagen. Restriction endonucleases, modifying enzymes, and Q5 High-Fidelity DNA polymerase, and deoxynucleotides were from New England Biolabs. In-Fusion HD Cloning Kit was purchased from Clontech. Invitrogen 1 kb Plus DNA ladder and BODIPY 493/503 were from Thermo Fisher Scientific. Oligonucleotides, Triton X-100, protease inhibitors, and bovine serum albumin were from Millipore-Sigma. Carrier DNA for yeast transformation was from Clontech. Bradford protein assay reagent, protein size standards, and electrophoretic reagents were from Bio-Rad. Radiochemicals were from PerkinElmer Life Sciences. Phospholipids were from Avanti Polar Lipids. Liquid scintillation mixtures were from National Diagnostics. Silica gel 60 TLC plates were from EMD Millipore.

### Strains and growth conditions

The bacterial (*E*. *coli*) and yeast (*S*. *cerevisiae*) strains used in this work are listed in Table 1. Yeast cells were grown at 30 °C in YEPD medium (1% yeast extract, 2% peptone, 2% glucose) or SC medium containing 2% glucose or galactose. The cell numbers of liquid cultures were estimated from spectrophotometric absorbance at 600 nm (A_600_). Cells were grown in SC to the exponential phase (A_600_ = 0.5, ∼12 h), and then grown for an additional 24 h to reach the stationary phase. For galactose induction, cells were grown for ∼14 h in the induction medium. Yeast transformants containing plasmids were cultured in an SC medium lacking appropriate nutrients. *E. coli* DH5α used for plasmid maintenance and amplification was grown at 37 °C in LB medium (1% tryptone, 0.5% yeast extract, 1% NaCl, pH 7.4). *E. coli* transformants containing plasmids were cultured in LB medium containing ampicillin (100 μg/ml).

**Table 1:** Strains and plasmids used in this study

Strain or plasmid	Relevant characteristics	Source or Ref
Strain		
*E. coli*		
DH5α	F^-^ φ80d*lacZ*ΔM15 Δ(*lacZYA*-*argF*)U169 *deoR recA1 endA1 hsdR17*(*r*_k_^-^*m*_*k*_^+^) *phoA supE44* λ^−^*thi-1 gyrA96 relA1*	(58)
*S. cerevisiae*		
RS453	*MAT***a***, ade2-1 his3-11,15 leu2-3112 trp1-1 ura3-52*	(59)
SS1026	*pah1*Δ*::TRP1* derivative of RS453	(24)
SS1132	*pah1*Δ*::TRP1 nem1*Δ*::HIS3* derivative of RS453	(15)
W303-1A	*MAT***a***ade2-1 can1-100 his3-11,15 leu2-3112 trp1-1 ura3-1*	(60)
GHY66	*app1*Δ*::natMX4 dpp1*Δ*::TRP1/Kan*^*r*^*lpp1*Δ*::HIS3/Kan*^*r*^*pah1*Δ*::URA3* derivative of W303-1A	(5)
Plasmid		
pRS415	Single-copy *E. coli*/yeast shuttle vector with *LEU2*	(61)
pGH315	*PAH1* gene inserted into the XbaI/HindIII sites of pRS415	(15)
pGH315-CR	pGH315 derivative for expression of Pah1-CR (Pah1 lacking residues 105–346, 592–628, and 646–834)	(28)
pGH315-CC	pGH315-CR derivative for expression of Pah1-CC (Pah1 lacking residues 105–346, 592–628, and 646–862)	This study
pYES2	High-copy *E. coli*/yeast shuttle vector with *URA3* and the *GAL1* promoter	Thermo Fisher Scientific
pGH452	Galactose-inducible expression of Pah1 with the C-terminal protein A tag	(27)
pGH465	Galactose-inducible expression of Pah1-CC with the C-terminal protein A tag	This study
pGH473	Galactose-inducible expression of Pah1-CR with the C-terminal protein A tag	This study
pGH316	pRS415 derivative containing the *PAH1* gene with the N-terminal HA tag	(26)
pGH319	pGH316 derivative containing human *HA-LPIN1*γ coding sequence under the control of the *PAH1* promoter	This study
pGH319-ΔHA	pGH315 derivative containing human *LPIN1*γ coding sequence	This study
pGH319-CC-ΔHA	pGH319-ΔHA derivative for expression of human lipin1 γ-CC (lipin 1γ lacking residues 109–649)	This study
YCplac33-*SEC63-GFP*	*SEC63*-*GFP* inserted into the *CEN/URA3* vector	(62)

### Plasmid constructions

Plasmids used in this study are listed in Table 1. Plasmid pGH315-CC (pRS415+*PAH1-CC*) was constructed from pGH315-CR (pRS415+*PAH1-CR*) by deleting the *PAH1* codons 647 to 862. Plasmid pGH465 (pYES2+*PAH1-CC*-*PtA*) was constructed from pGH452 (pYES2+*PAH1*-*PtA*) by replacing the *PAH1* sequence with the *PAH1-CC* sequence at the KpnI and EcoRI sites. Plasmid pGH473 (pYES2+*PAH1-CR-PtA*) was constructed from pGH452 (pYES2+*PAH1*-*PtA*) by replacing the SacI-NotI fragment of *PAH1-PtA* with the SacI-BstBI fragment of *PAH1-CR* from pGH315-CR and the BstBI-NotI fragment of *PtA* from pGH452. Plasmid pGH319 (pRS415+*HA*-*LPIN1*γ) was constructed from pGH316 (pRS415+*HA-PAH1*) by replacing the *HA-PAH1* sequence with the *HA-LPIN1*γ coding sequence at the AatII and EcoRI sites. Plasmid pGH319-ΔHA was constructed from pGH319 by deletion of the HA sequence. Plasmid pGH319-CC was constructed from pGH319 by deletion of the IDR sequence (codons 109–649). Plasmid pGH319-CC-ΔHA was constructed from pGH319-CC by deletion of the HA sequence.

### Preparation of yeast cell extracts, cytosol, and membranes

All steps were performed at 4 °C. Yeast cells were suspended in 50 mM Tris-HCl (pH 7.5), 0.3 M sucrose, 10 mM 2-mercaptoethanol, 0.5 mM phenylmethanesulfonyl fluoride, 1 mM benzamidine, 5 μg/ml aprotinin, 5 μg/ml leupeptin, and 5 μg/ml pepstatin. The cells were disrupted with glass beads (0.5 mm diameter) using a Biospec Products Mini-BeadBeater-16 (4). Unbroken cells and glass beads were removed by centrifugation at 1500*g* for 10 min. The resulting cell extract was centrifuged for 1 h at 100,000*g* to separate the cytosol (supernatant) from the membranes (pellet). The membrane pellet was resuspended in the same buffer used for cell disruption. Protein concentration was estimated by the method of Bradford (53) using bovine serum albumin as a standard.

### Purification of Pah1 and Pah1-CC

Protein A-tagged Pah1 and Pah1-CC were expressed in *S. cerevisiae* and purified according to the procedure described previously (27). Briefly, the fusion proteins were expressed through galactose induction in the *pah1*Δ *nem1*Δ strain (SS1132), which lacks the Pah1 phosphatase (*i.e.*, Nem1-Spo7 complex), and purified by affinity chromatography with IgG-Sepharose. The fusion proteins were cleaved with TEV protease to remove protein A, followed by anion exchange chromatography with Q-Sepharose (or strong ion exchange spin column). The purified preparations of Pah1 and Pah1-CC were stored at −80 ^°^C in the presence of 10% glycerol.

### Phosphorylation site analysis of Pah1-CC and identification peptide sequences by LC-MS/MS

The sites endogenously phosphorylated on purified Pah1-CC were analyzed by LC-MS/MS at the Center for Integrative Proteomics Research at Rutgers University as described by Park *et al.* (27). Details on the digestion of Pah1-CC in polyacrylamide gel slices with trypsin, chymotrypsin, or Glu-C and analysis of peptide fragments by LC-MS/MS are described previously (27). To confirm the identities of Pah1-CC, Sse2, Ssa1, and Hsp60 as the proteins contained within an SDS polyacrylamide gel slice was digested with trypsin at 37 °C followed by the analysis of the digest by LC-MS/MS (27). The raw data and database results for the peptide analyses of the proteins are presented in Table S1 and deposited in the MassIVE repository.

### PAP assay

PAP activity was measured as the amount of water-soluble ^32^P_i_ produced from chloroform-soluble [^32^P]PA as described by Carman and Lin (54). The reaction mixture consisted of 50 mM Tris-HCl (pH 7.5), 1 mM MgCl_2_, 0.2 mM PA (5000–10,000 cpm/nmol), 2 mM Triton X-100, and enzyme source in a total volume of 0.1 ml. The enzyme assay was conducted in triplicate at 30 °C for 20 min. The reaction was linear with time and protein concentration, and the average standard deviation of the assay was ± 5%. One unit of PAP activity was defined as the amount of enzyme that catalyzes the production of 1 nmol of phosphate per minute at 30 °C.

### Lipid labeling and analysis

Steady-state labeling of lipids with [2-^14^C]acetate was performed as described previously (4). Lipids were extracted from radiolabeled cells by the method of Bligh and Dyer (55). Neutral lipids were analyzed by one-dimensional thin-layer chromatography on silica gel plates using the solvent system hexane/diethyl ether/glacial acetic acid (40:10:1, v/v) (56). The identity of the labeled lipids on thin-layer chromatography plates was confirmed by comparison with standards after exposure to iodine vapor. Radiolabeled lipids were visualized by phosphorimaging analysis and quantified using ImageQuant software.

### Microscopy

For nuclear/ER membrane morphology analysis, yeast cells expressing the Sec63-GFP were grown in a synthetic selection medium at 30 °C to the exponential phase, and examined for the GFP signal by fluorescence microscopy. For the analysis of lipid droplets, the cells were grown to the stationary phase, stained for 30 min with 2 μM BODIPY 493/503, and observed by fluorescence microscopy. The average number of cells with normal nuclear/ER membrane structure (*i.e.* round-to oval-shaped circle) or the number of lipid droplets per cell was scored from ≥3 fields of view (≥150 cells). The fluorescence images were observed under a microscope (Nikon Eclipse Ni-U) with a long-pass green fluorescent protein filter, captured by the DS-Qi2 camera. All fluorescence images were analyzed with NIS-Elements BR software.

### Data analysis

Kinetic data were analyzed according to the Hill equation and Michaelis-Menten graph by the enzyme kinetics module of SigmaPlot software. Statistical analyses were performed with Microsoft Excel software. *p* values < 0.05 were taken as a significant difference.

## Data availability

Raw MS phosphorylation data and database search results for Pah1-CC, Sse2, Ssa1, and Hsp60 are deposited in the MassIVE repository (https://massive.ucsd.edu/ProteoSAFe/static/massive.jsp) with the accession number MSV000093075. All other data are contained within the manuscript or the supporting information.

## Supporting information

This article contains supporting information.

## Conflict of interest

The authors declare that they have no conflicts of interest with the contents of this article.
